# A complete genome sequence for *Pseudomonas syringae* pv. *pisi* PP1 highlights the importance of multiple modes of horizontal gene transfer during phytopathogen evolution

**DOI:** 10.1111/mpp.12806

**Published:** 2019-05-22

**Authors:** David A. Baltrus, Meara Clark

**Affiliations:** ^1^ School of Plant Sciences University of Arizona Tucson AZ 85721 USA; ^2^ School of Animal and Comparative Biomedical Sciences University of Arizona Tucson AZ 85721 USA

## Abstract

Hybrid assembly strategies that combine long‐read sequencing reads from Oxford Nanopore's MinION device combined with high‐depth Illumina paired‐end reads have enabled completion and circularization of both plasmids and chromosomes from multiple bacterial strains. Here we demonstrate the utility of supplementing Illumina paired‐end reads from a previously published draft genome of *P. syringae* pv. *pisi* PP1 with long reads to generate a complete genome sequence for this strain. The phylogenetic placement and genomic repertoire of virulence factors within this strain provides a unique perspective on virulence evolution within *P. syringae* phylogroup 2, and highlights that strains can rapidly acquire virulence factors through horizontal gene transfer by acquisition of plasmids as well as through chromosomal recombination.

We report the sequencing and assembly of a complete genome sequence for *Pseudomonas syringae* pv. *pisi* strain PP1. *P. syringae* phylogroup 2 strains usually contain a relatively low number of type III effectors in addition to multiple phytotoxins. Strain PP1 lacks (and likely has lost) these phytotoxins, and has regained numerous effectors compared to close relatives. This sequence extends a previously published analysis of virulence factors within this strain and enables investigation of the mechansisms of acquisition for new effector families. We find that these effectors were acquired through horizontal transfer of multiple plasmids, an integrative and conjugative element (ICE), a transposon, and likely through recombination of chromosomal regions.

Recent years and many draft genomes have provided a much needed comparative genomic framework for understanding phytopathogen evolution(Baltrus *et al*., 2017; Dillon *et al*., 2019b). Such studies have clearly demonstrated that rapid evolution of bacterial phytopathogens relies heavily on recombination and horizontal gene transfer of virulence factors. However, the strength of interpretations about the processes underlying these transfers remains somewhat limited because draft genome assemblies often lack important structural information. Thus, although comparative studies show that phytopathogen evolution is driven by horizontal transfer of phage, plasmid and chromosomal sequences, there is often a paucity of data concerning the importance of each mode of transfer as well as few clear links between how the prevalence of these events relates to the time scales being studied. Completing these genome sequences by supplementing them with long reads is a powerful and increasingly inexpensive way to gather this structural information.

*Pseudomonas syringae* phylogroup 2 strains can be isolated as pathogens from a range of different host plants, are found relatively often during sampling of host plants in the absence of disease as well as from other environmental sources and overall harbour a reduced set of type III effectors compared to other *P. syringae* phylogroups (Baltrus *et al*., 2011, 2014; Dillon *et al*., 2019a). This lack of effectors is correlated with the presence of phytotoxins such as syringolin, syringomycin and syringopeptin, which are conserved widely throughout this phylogroup (Dillon *et al*., 2019b). Correlations between phytotoxin acquisition and effector loss have sparked the idea that phytotoxins could be carrying out virulence functions that render effectors useless, either through phenotypic redundancy during infection or through alterations in ecological mechanisms of virulence (Baltrus *et al*., 2014; Hockett *et al*., 2014). Pathovar *pisi* strains are usually found as pathogens of peas (*Pisum sativum*) and are classified phylogenetically into *P. syringae* phylogroup 2 (Baltrus *et al*., 2014; Hollaway *et al*., 2007; Kraft and Pfleger, 2001). In contrast to other closely related pathovars and strains, phylogenetic analyses suggest that some pv. *pisi* strains have subsequently lost the capability to produce all three phytotoxins and have gained back numerous type III effectors through horizontal gene transfer (Baltrus *et al*., 2011, 2014). Indeed pv. *pisi* strains have one of the the highest number of predicted type III effectors of any of the sequenced phylogroup 2 strains (Dillon *et al*., 2019a). Since draft genomes of previously sequenced pv. *pisi* strains have been assembled using only ‘short read’ technologies, questions remain concerning how these effectors were acquired over relatively short periods of evolutionary time in the context of *P. syringae* evolution writ large. Were all effectors rapidly gained through acquisition by horizontal gene transfer of plasmids or prophage, or were some gained by recombination of chromosomal regions? These questions would easily be answered by linking genome structure with effector presence, which is enabled by assembly of a complete genome sequence for a pv. *pisi* strain.

Here we report the complete genome sequence of *P. syringae* pv. *pisi* PP1 (MAFF301208), originally isolated as a pathogen of sweet pea. We originally reported assembly of a draft genome for this strain using Illumina paired‐end reads (Baltrus *et al*., 2014), but have now completed this genome by employing a hybrid approach involving long reads from an Oxford Nanopore MinION. Access to this complete genome sequence, which consists of one chromosome and three plasmids, enables definitive placement of type III effectors onto replicons and therefore allows investigation into how these effectors were acquired. Using this genome sequence we therefore can demonstrate that, even though numerous type III effectors have been acquired in concert with loss of at least two of three phytotoxins present in other group II strains, horizontal transfers of these effectors have taken place through recombination of chromosomal segments as well through plasmid acquisition. These results and this complete genome sequence provide important context for understanding the mechanisms behind how phytopathogens acquire new virulence genes, but also enable further exploration of evolutionary and ecological pressures that provide structure to toxin and effector content across *P. syringae*.

*Pseudomonas syringae* pv. *pisi* was originally acquired from the culture collection of Japan's Ministry of Agriculture, Forestry, and Fisheries (MAFF301208). We have originally reported a draft genome sequence for this strain, using only 100 bp paired‐end reads from an Illumina HiSeq, which consisted of an assembly of 5 949 520 bp spread over 256 contigs (Baltrus *et al*., 2014). For original Illumina sequencing, a single colony of this strain was isolated on King's B (KB) media and grown overnight in 2 mL of liquid KB media. The totality of this culture was pelleted for DNA extraction using a Genepro Wizard Kit, including RNAse addition. Genomic DNA was independently extracted using these same procedures for sequencing using the Oxford Nanopore MinION. Genomic DNA from strain PP1 was mixed with genomic DNA from three other strains (*P .syringae* strain GAW0119, DBL542; *Frondihabitans* sp. 14F, DBL1621; and *Pantoea* sp. 4aiii, DBL1298), and the entirety of this DNA pool was sequenced on an R9.4 flowcell using a single preparation from a Rapid sequencing kit (SQK‐RAD004). In total, 270 486 reads were generated on the MinION device for this mixed run. A genome assembly was created by combining both Illumina and MinION reads using Unicycler (version 0.4.4) with default parameters. For the PP1 assembly, 19 707 of the 270 486 reads (105 683 222 bp total aligned, 5362 bp average length per read aligned) MinION reads were able to be fully or partially aligned to the genome sequence. We tested whether including mixed DNA samples in the long‐read library above erroneously introduced additional sequences from the other strains into the assembly for strain PP1 by mapping back Illumina reads to the complete genome sequence using Geneious Prime (version 2019.0.4, mapped using default parameters). Every base pair of the chromosome and from all of the plasmids was supported by at least 40× coverage of Illumina reads, thereby supporting the idea that no erroneous sequences were introduced by the inclusion of the mixed MinION reads.

To further investigate the potential that inclusion of the mixed MinION reads above led to erroneous assembly of the PP1 genome, we performed a second assembly using the Illumina reads from above combined with independently sequenced genomic DNA from *P. syringae* pv. *pisi* PP1 using the MinION. For this library, genomic DNA was prepped as above, independently from other genomic preps, and sequenced using the Rapid Barcoding kit (SQK‐RBK004). Reads from this MinION run labelled with barcode number 7 were subsequently indexed and trimmed using Porechop (Wick, 2017b). For this second PP1 assembly, 178 850 MinION reads (426 412 513 bp total aligned, 2384 bp average length per read) were mapped to contigs generated by the Illumina reads. We note that this assembly was generally of poorer quality despite additional depth (30 contigs, but approximately the same total size as the publically reported genome) than that sequenced using the mixed DNA prep, likely because the libraries prepared for the first assembly contained longer reads (and especially more reads >20 kb). We have mapped these contigs back to the complete genome assembly available at Genbank using Geneious Prime (version 2019.0.4, mapped using default parameters). Mapping of these contigs demonstrates that both assemblies are syntenic and that there are no large‐scale additions or deletions of genes between the two, but that the second assembly breaks down at regions of insertion sequence (IS) elements and across highly similar regions of plasmids pPP1‐1 and pPP1‐2. Indeed, there are multiple long (>20 kb) reads from the mixed sequencing library that cover these regions completely (data not shown). We have included a fasta file containing this assembly as a file on Figshare as well as the Unicycler log for this second assembly (https://figshare.com/s/4e2fdb486d1c31375b52).

Unicycler (Wick *et al*., 2017) assembly of the mixed Nanopore library and the Illumina reads using default parameters yielded three circularized plasmids and two contigs that we hypothesized would together represent the circularized chromosome. To demonstrate circularization of these contigs, we designed three primer pairs (DBL646 (ATCCCGAACGAGATGAAGCC) and DBL647 (CGACCGCTCTTTTACTCAGACCAG), DBL648 (AAACCAGCATCCCGAACGAG) and DBL649 (TGGACCCAATCCTTCATCCG), DBL657 (CGGCGTGAACAAATGCTTTAGATG) and DBL658 (CCTGGTGAAAACTCAGTGTATTGGG)) that would amplify off the ends of each of the contigs if they were both part of a circular chromosome. PCR using a standard protocol (55 °C annealing temperature and  a 1 min 30 s extension time) with genomic DNA and these primer sets yielded clear bands of the estimated size, and sequencing of these PCR products definitively showed that both remaining contigs were part of the same circular chromosome. Files from Sanger sequencing of these regions can be found at https://figshare.com/s/4e2fdb486d1c31375b52.

This updated genome sequence replaced the previous draft genome at Genbank and is found under accession numbers CP034078‐CP034081. Raw reads from all sequencing platforms are available at the SRA database under BioProject accession PRJNA211606. MinION reads from the mixed library are available in the SRA database at accession SRR8767346, MinION reads from the PP1 alone library are available at accession SRR8754885, while Illumina reads are available at accession SRR8412066. This complete genome was independently annotated using NCBI's PGAP pipeline (Tatusova *et al.*, 2016).

The complete nucleotide sequence of the chromosome was submitted to the PHAST server (http://phast.wishartlab.com/index.html; Zhou *et al*., 2011) to identify putative phage regions using default parameters. A file containing annotation results fom the PHAST program for the PP1 genome can be found at https://figshare.com/s/4e2fdb486d1c31375b52.

A file containing amino acid sequences of known type III effectors from *P. syringae,* available at https://figshare.com/s/4e2fdb486d1c31375b52, was used to query the complete genome sequence of strain PP1 using the program tblastn (‐e 1e‐5, ‐outfmt 6). This list was created by taking single sequences from all effector families and subfamilies from the curated database found at www.pseudomonas-syringae.org. The placement of each potential effector hit onto either the chromosome or one of the plasmids was noted within the blast results. Since some type III effectors are chimeras of two different effector proteins, potential ‘hits’ were vetted for positional overlap with other potential matches by hand from the blast results. Given the relatively broad search parameters, and no direct screening for protein length, effectors would show up in the blast results as partial hits from multiple effector families at very close proximity within the genome. However, there appear to be no clear instances of new chimeric effectors within this genome. Nucleotide sequences of each potential effector were then extracted from the genome by hand so that the sequence extended from the first in‐frame stop codon 5′ to the effector hit to the next viable in‐frame stop codon 3′ to the effector hit. Nucleotide sequences of potential effectors were then trimmed so that start codons (likely ATG, but sometimes GTG) positionally matched other members from the same effector family or were left unaltered with the notation that effectors lacked a clearly identifiable start codon and may therefore not be functional. Potential effectors, as well as fragments of known effectors displaying high similarity in Blast comparisons but which were either lacking start codons or which were significantly truncated, were also flagged as possible effectors. A file containing the effector annotations and placement can be found at https://figshare.com/s/4e2fdb486d1c31375b52.

Within this figshare data set we have also included a file containing select protein sequences from known toxin pathways within *P. syringae*. As with the effector sequences above, this file was used to query the PP1 genome using tblastn (evalue ‐1e5) to evaluate the presence of pathways potentially encoding phytotoxins. As previously reported (Baltrus *et al.*, 2014), and with the exception of the truncated 3′ end of *sypC*, there are no hits to the syringolin, syringomycin or syringopeptin pathways in strain PP1.

A hybrid assembly approach, combining short paired‐end Illumina reads with longer reads from an Oxford Nanopore MinION, was used to create a whole genome assembly for *P. syringae* pv. *pisi* strain PP1. This assembly is significantly improved over the previously reported draft genome for the same strain (Baltrus *et al*., 2014) and enables clear delineation of the genome into four distinct circular replicons: one chromosome (5 883 416 bp) and three plasmids (pPP1‐1, 62 150 bp; pPP1‐2, 54 993 bp; pPP1‐3, 39 003 bp). Furthermore, a complete genome sequence also enables identification of one complete prophage (from bp positions 3 514 617 to 3 545 192) on the chromosome of this strain.

Having a complete genome sequence enables a comprehensive annotation of the type III effector catalogue for this strain and provides the structural and mechanistic context for how those effectors were acquired. As one can see from Fig. 1, even though pathovar *pisi* strains have one of the highest number of effectors across phylogroup II strains (Dillon *et al*., 2019a), many of these effectors are shared with other phylogroup 2 strains with slightly smaller repertoires. How did pathovar *pisi* acquire extra effectors compared to other group II strains? At least four of the potential effectors were acquired in two independent steps through conjugation of two plasmids (*hopAF‐*like and *hopAM1* on pPP1‐1; *avrRps4/hopK* and *hopR1* on pPP1‐2). *shcF/hopF1* also appears to have been acquired by horizontal gene transfer as it appears to be present within a transposon. *hopC1* is found in close association with *hopH1* within the genome of strain PP1, even though they are present at fairly different frequencies in phylogroup 2 strains. The genome sequence surrounding these genes (~10 kb downstream) contains genes annotated as conjugal transfer proteins and thus it is likely that these effectors were acquired through conjugation as part of an ICE element. The mechanism of acquisition of *hopE1* remains unclear as it is not present on a plasmid or associated with mobile elements like transposons or prophage. It is therefore possible that this effector was acquired through recombination, although the mechanism behind such events, frequent as they may be, is unknown.

**Figure 1:**
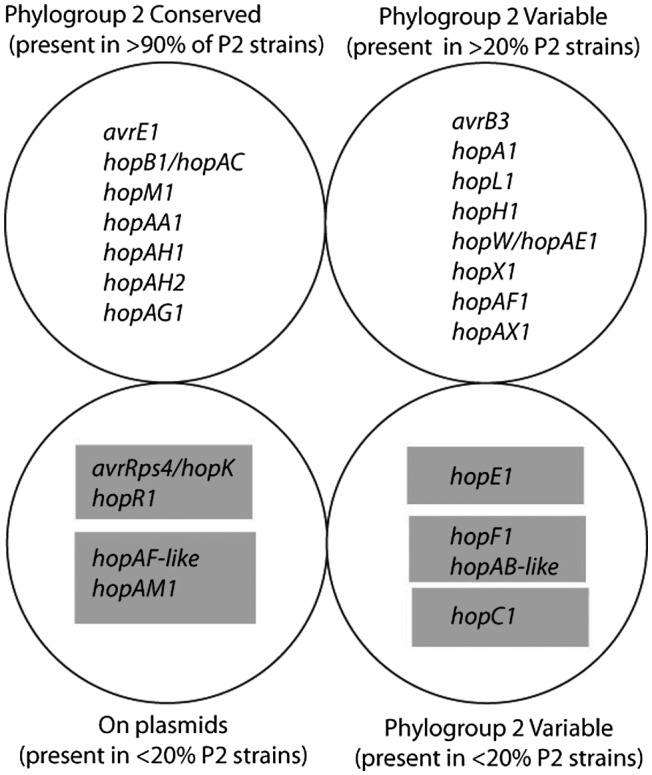
Effector proteins in pathovar *pisi* have been horizontally acquired through multiple routes. Each predicted type III effector protein within the genome of strain PP1 was placed onto a replicon. Since each plasmid‐based effector is relatively rare in the phylogroup and is present in <20% of phylogroup 2 strains according to (Dillon *et al.*, 2019b), we have pulled out chromosomal effectors that could also be considered rare. Relatively rare effectors that were horizontally acquired and only present in strain PP1 were further grouped by structural linkage on either the same plasmid or by proximate position on the chromosome (indicated by grey boxes). Strain PP1 has acquired four predicted effectors through two separate plasmid acquisitions and four other potential effectors through chromosomal recombination. Lastly, we note that loci resembling the C terminus of *hopAT1* and the N terminus of *hopAQ1* are present within the genome, but it is truncated and divergent and so we hesitate to include it in this list.

Recharacterization of the newly assembled complete genome sequence for strain PP1 also enables an investigation into the genomic basis for the loss of all three toxins normally found within phylogroup II. Strain PP1 contains no close BlastP hits for a majority of the genes involved in syringolin biosynthesis. However, analysis of the genomic context syntenic to upstream and downstream regions of the syringolin biosynthesis pathway in B728a does contain an ortholog (RS020135) to Psyr_1701 (Fig. 2). However, this locus is followed by two genes annotated as a hypothetical gene and a PAAR domain‐containing protein that do not have close matches within the B728a genome, which replace the syringolin biosynthesis pathway (Psyr_1701‐Psyr_1706). Finally, synteny is re‐established downstream of these genes as strain PP1 contains numerous downstream orthologs beginning with a match to Psyr_1707. Further genomic comparisons using BlastP demonstrate that this region within strain PP1 is actually highly syntenic to that of *P. syringae* pv. *syringae* strain SM (Dudnik and Dudler, 2013). Strain SM groups within phylogroup II, contains a highly reduced effector repertoire and does not naturally produce syringolin. It is therefore clear that presence of pathways involved in syringolin biosynthesis do not strictly correlate with the size of the effector repertoire.

**Figure 2:**
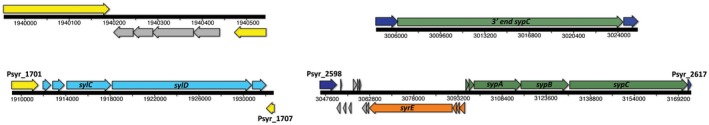
Comparison of synteny between phytotoxin loci in strain PP1 and *Psy*B728a. Genomes of strains PP1 and pv. *syringae* B728a were queried for synteny in loci surrounding phytotoxins syringolin, syringomycin and syringopeptin, with structural results shown in the figure. Anchor points for synteny comparisons and which are present within both genomes are coloured to represent shared presence (syringolin, yellow *Psyr_*1701 and *Psyr_1707*; syringomycin/syringopeptin, blue *Psyr*_2598 and *Psyr*_2617). Genes involved in syringolin production are sky blue, those involved in syringomycin production are orange and those involved in syringopeptin production are green. Genomic locations within each annotation genome are also represented by the red numbers below locus representations.

Genes involved in the production of syringomycin and syringopeptin are located in close proximity to each other in the genome of *P. syringae* pv. *syringae* strain B728a (Fig. 2). Both PP1 (RS0206540) and B728 (Psyr_2598) contain highly similar genes annotated as a DNA helicase and/or a DUF4011‐containing protein in the same genomic context upstream of the syringomycin/syringopeptin toxin cluster, except that the PP1 helicase is predicted to be 1350 amino acids shorter than the B728a version. The downstream region of this helicase in strain PP1 contains one long gene predicted to be involved in amino acid adenylation and which closely matches the last half of Psyr_2616, which encodes the last gene involved in the syringopeptin biosynthetic pathway (*syrC*). The region downstream of this amino acid adenylation gene in PP1 also re‐establishes complete synteny with the B728a chromosome, suggesting that the region encoding syringomycin and syringopeptin as well as numerous other loci was cleanly deleted in an ancestor of strain PP1 leaving a genomic scar containing orthologs of the the front half of Psyr_2598 and the 3′ end of *sypC/*Psyr_2616 (Fig. 2B).

A complete genome sequences for strain PP1 enables a revisitation of the previously annotated type III effector repertoire (Baltrus *et al*., 2014) compared to what was identified from the draft genome alone. Comparison of effectors from both iterations of this genome sequence are nearly identical, and thus give confidence that effector annotations within draft genomes are a reasonable approximation of the genes that are present. However, there are a handful of slight discrepancies that came to light with reannotation of the complete genome sequence. In the published draft genome of strain PP1, we predicted that this strain would harbour the effector *hopI*, which is widely conserved across *P. syringae* strains and is thought to disrupt chloroplast functions. However, it is now clear that *hopI* is actually missing from the genome of PP1 and that previous annotation mischaracterized the *P. syringae dnaJ* gene that shares sequence similarity with *hopI*. Likewise, having structural information from a complete genome in hand creates a higher confidence in a prediction that a new effector family is present in a genomic island with *hopF1.* The sequence of this locus is similar to that of *hopAB/hopAY*, but it displays enough divergence that we hesitate to call it a new effector family at present without additional data. Lastly, one of the plasmids also contains a locus that is nearly sequence identical to the C terminus of the *Xanthomonas* effector family (AvrXv3 or XopAF1), the nomenclature of which is due itself to sequence similarity with HopAF1. It is clear that this locus is separate from the annotated *hopAF1* gene within this genome, both in genomic location and sequence, and thus it is worth further characterization as a virulence factor. We also note that since this locus is present on a plasmid, and is highly similar to genes found within Xanthomonas, it emphasizes the potential for horizontal gene transfer of virulence factors to occur across phytopathogens.

Lastly, the ability to delineate individual replicons within this genome sequence enables sequence comparisons across the replicons themselves. One interesting result from such comparisons is that plasmids pPP1‐1 and pPP1‐2 are nearly sequence identical for a significant (one single nucleotide variant over 17 348 bp, from bp 30 846 to bp 48 193 on pPP1‐1) portion of their length even though the rest of the plasmids are quite divergent. This region is also a close match (94% nucleotide similarity) for a 13 kb region on plasmid p3_tig5 in pathovar *morsprunorum* strain R15244. Genes within this region are annotated as parts of a type IV secretion system and so it is likely that this region controls conjugation of these plasmids across strains. We highlight this region because it is particularly useful to note that the hybrid assembly strategy clearly and independently allowed for assembly of each replicon despite this high sequence similarity.

In summary, the complete genome sequence for *P. syringae* pv. *pisi* PP1 enables finer scale structural analyses that highlight how virulence factors in phytopathogens can be acquired over relatively short periods of time. Although the effects of horizontal gene transfer on bacterial evolution have been well known for decades, sequencing of this particular strain provides insights within a unique evolutionary framework where genomic trends in one particular lineage of *P. syringae* phylogroup 2 run counter to overriding trends for a variety of closely related strains. Overall, although virulence genes have been gained through the acquisition of two different plasmids, some virulence genes have also been acquired through recombination with chromosomal regions partially mediated by movement of transposons.
